# Bone metabolism genes variation and response to bisphosphonate treatment in women with postmenopausal osteoporosis

**DOI:** 10.1371/journal.pone.0221511

**Published:** 2019-08-22

**Authors:** Pavel Marozik, Vidmantas Alekna, Ema Rudenko, Marija Tamulaitiene, Alena Rudenka, Asta Mastaviciute, Volha Samokhovec, Andrejus Cernovas, Katsiaryna Kobets, Irma Mosse

**Affiliations:** 1 Laboratory of Human Genetics, Institute of Genetics and Cytology of the National Academy of Sciences of Belarus, Minsk, Belarus; 2 Department of General Ecology, Biology, and Environmental Genetics, International Sakharov Environmental Institute of the Belarusian State University, Minsk, Belarus; 3 Faculty of Medicine, Vilnius University, Vilnius, Lithuania; 4 Department of Cardiology and Internal Diseases, Belarusian State Medical University, Minsk, Belarus; 5 Department of Cardiology and Rheumatology, Belarusian Medical Academy of Post-Graduate Education, Minsk, Belarus; Van Andel Institute, UNITED STATES

## Abstract

**Introduction:**

Long-term treatment is used in patients with osteoporosis, and bisphosphonates (BPs) are the most commonly prescribed medications. However, in some patients this therapy is not effective, cause different side effects and complications. Unfortunately, at least one year is needed to identify and confirm an ineffectiveness of BPs therapy on bone mineral density (BMD). Among other factors, a response to BPs therapy may also be explained by genetic factors. The aim of this study was to analyze the influence of *SOST*, *PTH*, *FGF2*, *FDPS*, *GGPS1*, and *LRP5* gene variants on the response to treatment with aminobisphosphonates.

**Materials and methods:**

Women with postmenopausal osteoporosis were included to this study if they used aminobisphosphonates for at least 12 months. Exclusion criteria were: persistence on BPs therapy less than 80%, bone metabolic diseases, diseases deemed to affect bone metabolism, malignant tumours, using of any medications influencing BMD. The study protocol was approved by the local ethics committee. The BMD at the lumbar spine and femoral neck were measured using dual x-ray absorptiometry (GE Lunar) before and at least 12 months after treatment with BPs. According to BMD change, patients were divided in two groups–responders and non-responders to BPs terapy. Polymorphic variants in *SOST*, *PTH*, *FGF2*, *FDPS*, *GGPS1*, and *LRP5* genes were determined using PCR analysis with TaqMan probes (Thermo Scientific).

**Results:**

In total, 201 women with BPs therapy were included in the study. No statistically significant differences were observed in age, age at menopause, weight, height, BMI and baseline BMD levels between responders (122 subjects) and non-responders (79 subjects).

As single markers, the *SOST* rs1234612 T/T (OR = 2.3; P = 0.02), *PTH* rs7125774 T/T (OR = 2.8, P = 0.0009), *FDPS* rs2297480 G/G (OR = 29.3, P = 2.2×10^−7^), and *GGPS1* rs10925503 C/C+C/T (OR = 2.9; P = 0.003) gene variants were over-represented in non-responders group. No significant association between *FGF2* rs6854081 and *LRP5* rs3736228 gene variants and response to BPs treatment was observed. The carriers of T-T-G-C allelic combination (constructed from rs1234612, rs7125774, rs2297480, and rs10925503) were predisposed to negative response to BPs treatment (OR = 4.9, 95% CI 1.7–14.6, P = 0.005). The C-C-T-C combination was significantly over-represented in responders (OR = 0.1, 95% CI 0.1–0.5, P = 0.006).

**Conclusions:**

Our findings highlight the importance of identified single gene variants and their allelic combinations for pharmacogenetics of BPs therapy of osteoporosis. Complex screening of these genetic markers could be used as a new strategy for personalized antiresorptive therapy.

## Introduction

Osteoporotic fragility fractures are the most dangerous complication of osteoporosis, leading to increased mortality, morbidity, and enormous social and medical expenses worldwide [1]. According to current prognosis, among older population one in three women and one in five men will suffer from osteoporotic fracture in later life, and this may result in serious medical consequences, loss of independence or death [2, 3]. Long-term antiosteoporotic treatment should be prescribed to patients with osteoporosis and high fracture risk to stabilize or increase bone mineral density and prevent fragility fractures. There are several approved medications available for this purpose: antiresorptive drugs (selective estrogen receptor modulators, bisphosphonates and denosumab) and anabolic agents derived from parathyroid hormone. All these therapies are effective in reducing the risk for vertebral osteoporotic fractures, while alendronate, risedronate, zoledronate and denosumab also reduce the risk for nonvertebral fractures, including hip fractures [4]. Bisphosphonates (BPs) are the most commonly used antiosteoporotic medications, approved by the US Food and Drug Administration (FDA) and the European Medicines Agency (EMA) for the treatment of postmenopausal osteoporosis. Current evidence suggest that long term use of BPs is effective for preserving bone mineral density and should be planned for patients with high fracture risk. Results for up to 10 years of treatment with alendronate [5], 7 years with risedronate [6] and 6 years with zoledronic acid [7] are available. However, prolonged use of BPs may lead to undesirable effects, such as osteonecrosis of jaw, atypical femoral fractures, atrial fibrillation and esophageal diseases [8]. Another important issue regarding long-term pharmacological therapy of osteoporosis is adherence to treatment. As it was shown in systematic review by Vieira *et al* [9], adherence to treatment of osteoporosis with BPs is influenced by many factors and remains inadequate, while monthly dosage of BPs was associated with greater adherence compared with weekly dosage. Given the above, physicians need to reassess the indication for continued use of BPs beyond 3 to 5 years considering their effectiveness, side effects and complications. According to the study of Clark *et al*, about 15% of older women receiving oral medications for osteoporosis had low adherence to prescribed treatment and this fact has important implications for healthcare provision [10]. Up to 53% of patients with osteoporosis exhibit poor response to BPs treatment [11].

On the other hand, osteoporosis has a strong genetic component and up to 80% of the variability of bone mineral density (BMD) may be due to hereditary factors. Thus, differences in response to BPs therapy may also be explained by genetic factors, to a certain extent. Identifying genes that may be associated with response to BPs is a challenging and highly promising task choosing an individual personalized therapy in patients with osteoporosis. A few recently published pharmacogenetic studies revealed some gene variants that could have a predictive value of response to BPs in osteoporosis [12–19]. However, these findings should be confirmed by further studies performed in a larger population and in different ethnic groups.

Single nucleotide variants (SNVs) were selected from genes, involved in regulation of bone remodeling processes and could be effective markers of BPs therapy, increasing BMD and reducing bone fracture risk in patients with postmenopausal osteoporosis. *SOST* and *LRP5* genes are the modulators of the *Wnt* signaling pathway, which is believed to play an important role in bone formation. Sclerostin inhibits osteoblast activity by binding to the LRP5/6 coreceptor and was previously shown to be associated with the response to alendronate treatment in Chinese women [12,13]. *FDPS* and *GGPS* genes are the important regulators of intracellular mevalonate pathway and are inhibited by BPs [13–15]. *PTH* and *FGF2* genes were included to the study because of their important role in bone remodeling [19,20].

The aim of this study was to analyze the influence of *SOST* (sclerostin), *PTH* (parathyroid hormone), *FGF2* (fibroblast growth factor 2), *FDPS* (farnesyl diphosphate synthase), *GGPS1* (geranylgeranyl diphosphate synthase), and *LRP5* (low density lipoprotein receptor-related protein 5) gene variants on the response to treatment with BPs.

## Materials and methods

### Subjects

This retrospective cohort study was conducted at out-patient clinics. White Caucasian women with established diagnosis of postmenopausal osteoporosis according to WHO Diagnostic Criteria for Osteoporosis [21], who used aminobisphosphonates (alendronate, risedronate, ibandronate or zoledronate) at least 12 months, were included into this study at Minsk City Center for Osteoporosis and Bone-Muscular Diseases Prevention (Minsk, Belarus) and in National Osteoporosis Center (Vilnius, Lithuania). All patients also used calcium and vitamin D supplements. Exclusion criteria were: persistence on BPs therapy less than 80%, presence of metabolic bone diseases (such as Paget's disease and osteomalacia), diseases deemed to affect bone metabolism (such as hyperthyroidism, renal failure, Cron’s disease, and rheumatoid arthritis), malignant tumors of various localizations, using of any medications likely to influence BMD (glucocorticoid therapy, anticoagulants). All subjects signed written informed consent after being fully informed about the nature of the study in accordance with the declaration of Helsinki (as revised in 2013). The study protocol was approved by the Local Research Ethics Committee at the Belarusian Medical Academy of Postgraduate Education, Local Ethics Committee at 1^st^ Minsk City Hospital and Lithuanian Regional Biomedical Research Ethics Committee.

### BMD measurement

BMD was evaluated by dual-energy x-ray absorptiometry (GE Lunar, Madison, WI, USA). The equipment was calibrated daily using a standard spine phantom provided by the manufacturer. Lumbar spine L1–L4 (LS) and femoral neck (FN) BMD (g/cm^2^) was measured on the same machine at baseline and at least after 12 months of treatment. The response to BPs therapy was evaluated according to the BMD trend in LS region, as it has the highest precision and the most rapid change in response to therapy compared with FN BMD [22]. An increase of LS BMD that exceeded the least significant change (LSC, determined as 3.1) calculated using standardized methods [23], was considered as acceptable response to therapy, while decrease in BMD was considered as treatment inefficiency. According to this, patients were classified as responders or non-responders to BPs therapy. Patients with the change of LS BMD that did not exceed the LSC were not included in further analysis.

### Genotyping

For genetic analysis, venous blood samples were taken from the cubital vein using the Vacutainer system with EDTA (Beckton-Dickinson, USA). DNA was isolated using the standard proteinase K digestion, phenol-chloroform extraction, and ethanol precipitation. The DNA solution was extracted with a phenol-chloroform-isoamyl alcohol mixture to remove protein contaminants and then was precipitated with 100% ethanol. The DNA was pelleted after the precipitation step, washed with 70% ethanol to remove salts and small organic molecules, and resuspended in a buffer at a concentration suitable for further investigation (20–120 ng/μL). The quantity of DNA samples was checked using Qubit 2 Fluorimeter (Thermo Scientific, USA), the quality and purity were checked using NanoDrop 8000 spectrophotometer (Thermo Scientific, USA).

Information on polymorphic variants of *SOST*, *PTH*, *FGF2*, *FDPS*, *GGPS1*, and *LRP5* genes was obtained from Entrez Gene database (www.ncbi.nlm.nih.gov/gene). Single nucleotide variants for the study were selected according to the following criteria: involvement in bone and bisphosphonate metabolism; minor allele frequency higher than 10% in Caucasian population. Selected markers (rs1234612, rs7125774, rs6854081, rs2297480, rs10925503, and rs3736228) were determined using the quantitative polymerase chain reaction (PCR) with TaqMan Probes (Thermo Fisher Scientific, USA). Briefly, the PCR reaction system consisted of 5 μL iTaq™ Universal Probes Supermix BioRad©, 3.75 μL of mQ water, 0.25 μL ×40 TaqMan™ SNP Genotyping Assays Applied biosystems©, 1 μL of genomic DNA (15 ng) to the PCR tubes. The PCR was performed with an initial denaturation at 95°C for 10 min, followed by 40 cycles of denaturation at 95°C for 15 s, annealing and synthesis at 60°C for 30 s, read fluorescence plates. The Real Time PCR amplification was carried out in the CFX96™ Real-Time PCR Detection Systems (Bio-Rad^©^, USA). The final extension was performed at 72°C for 1 min. Negative and positive controls were randomly included across each PCR run, several samples were randomly re-genotyped for quality control purposes.

### Statistical analysis

Kolmogorov-Smirnov test was used to assess the normality of data distribution. The data was not normally distributed, presented as median (25%, 75% interquartile range) and compared using Mann-Whitney U-test. The BMD trend (in %) was calculated as (BMD level after treatment–baseline BMD)/baseline BMD ×100%. The BMD trend (%, per year) represents the average annual percentage change of BMD in comparison to baseline BMD.

Based on the determined frequencies of genotypes and by using the Pearson chi-square (χ^2^) test, the Hardy-Weinberg equilibrium was assessed to detect genotyping errors. The risk of non-response to treatment was estimated using odds ratios, with 95% confidence intervals (CI) and calculated in comparison to reference (major homozygous) genotype. Additive genetic model was defined and tested for all SNVs, the dominant model was tested when the minor allele frequency (MAF) was 0.1 or lower. Logistic regression models were used to assess difference between the characteristics of responders and non-responders groups for categorical data and for comparison of genotype frequencies between these groups. Allelic combinations were constructed and analyzed for association with drug response using the R-package “SNPassoc” (v. 1.9–2), the programs used likelihood ratio tests in a generalized linear model and the expectation-maximization algorithm [24].

All statistical analyses were performed using the programming language R (available at http://r-project.org). The differences between the groups were considered statistically significant at P<0.05. Bonferroni corrections were conducted for multiple comparisons. For SNVs analysis, the Bonferroni-corrected P-value threshold for statistical significance was considered 0.0083.

## Results

In total, 357 postmenopausal women with BPs therapy were screened, of them 156 subjects were excluded as their change of LS BMD did not exceed the LSC and their adherence was less than 80%, and 201 subjects (of them 73 –Belarusian, 128 –Lithuanian) were included in the study according to inclusion criteria. Clinical characteristics of women with osteoporosis are presented in Table 1. The mean interval between BMD measurements was 2.1 years (1–2 years, 56.7%; 2–3 years, 37.8%; more than 3 years, 5.5% women). Aminobisphosphonates-treated women used alendronate (n = 47), risedronate (n = 34), ibandronate (n = 103) and zoledronate (n = 17). No statistically significant difference was revealed between responders and non-responders depending on the therapeutic agents (Table 1).

**Table 1:** Clinical characteristics of study subjects.

		Responders	Non-responders	P
Number (%)		122 (60.7%)	79 (39.3%)	-
Age, years		65.0 (60.0; 70.9)	64.0 (59.0; 68.0)	0.15
Age at menopause, years		50.0 (48.0; 52.0)	50.0 (48.0; 53.0)	0.95
Weight, kg		65.0 (60.0; 72.8)	62.0 (58.0; 71.0)	0.41
Height, cm		160.0 (156.0; 164.0)	159.0 (155.0; 163.5)	0.80
BMI		25.6 (22.7; 28.7)	25.2 (22.5; 28.5)	0.83
Amino bisphosphonate, n (%)	Alendronate	32 (68.1)	15 (31.9)	0.23
	Risedronate	21 (61.8)	13 (38.2)	0.89
	Ibandronate	57 (55.3)	46 (44.7)	0.11
	Zoledronate	12 (70.6)	5 (29.4)	0.37
Baseline LS BMD, g/cm^2^		0.85 (0.76; 0.90)	0.86 (0.83; 0.92)	0.11
LS BMD after treatment, g/cm^2^		0.91 (0.82; 0.97)	0.82 (0.78; 0.86)	8.6×10^−6^
LS BMD trend, %		6.0 (4.4; 8.4)	-3.7 (-4.3; -3.4)	2.2×10^−12^
LS BMD trend, % per year		3.4 (2.7; 5.1)	-2.3 (-3.4; -1.8)	2.2×10^−12^

The data is presented as mean (25%; 75% interquartile range)

In the study cohort, 201 patients had low BMD at spine of L1–L4 (T-score ≤-2.5 below the mean for young adult women); among them, 93 women had a fracture history (at least one). The average age of all the examined women was 64.0 (60.0; 70.0) years, age at menopause– 50.0 (48.0; 52.0) years, the average baseline BMD of L1–L4 and FN were 0.85 (0.79; 0.91) and 0.79 (0.71; 0.85) g/cm^2^, respectively. No statistically significant differences were observed in age, age at menopause, weight, height, BMI and baseline BMD levels between responders and non-responders. Statistically significant difference between responders and non-responders was revealed only for LS BMD level after treatment.

All patients were genotyped in the study. We did not reveal any statistically significant association of analyzed SNVs with baseline L1-L4 BMD level (not shown). The distribution of genotype frequencies of *SOST*, *PTH*, *FGF2*, *FDPS*, *GGPS1*, and *LRP5* gene variants together with the P-values are shown in Table 2.

**Table 2:** The Hardy-Weinberg equilibrium (HWE) P-values and distribution of genotype frequencies of *SOST*, *PTH*, *FGF2*, *FDPS*, *GGPS1*, and *LRP5* gene variants in responders and non-responders.

Gene variant	Genotype	Non-responders *n* = 79		Responders *n* = 122		OR (95% CI)	P-value
		%	HWE	%	HWE		
***SOST* rs1234612**	T/T	63.3	0.33	42.6	0.84	1	0.016
	C/T	30.4		46.7		0.4 (0.2–0.8)	
	C/C	6.3		10.7		0.4 (0.1–1.2)	
***PTH* rs7125774**	T/T	50.6	0.78	27.1	0.28	1	**0.0009**
	C/T	43.0		54.9		0.4 (0.2–0.8)	
	C/C	6.4		18.0		**0.2 (0.1–0.6)**	
***FGF2* rs6854081**	T/T	75.3	0.59	78.7	0.65	1	NS ^a^
	T/G	24.7		19.7		1.3 (0.7–2.6)	
	G/G	0.0		1.6		-	
***FDPS* rs2297480**	T/T	36.7	0.34	75.4	0.69	1	**2.2×10**^**−7**^ ^b^
	T/G	52.2		23.7		4.5 (2.4–8.7)	
	G/G	11.1		0.8		**29.3 (3.6–241.0)**	
***GGPS1***^***c***^ **rs10925503**	C/C	69.6	0.69	86.9	0.12	1	**0.003**
	C/T+T/T	30.4		13.1		**2.9 (1.4–5.9)**	
***LRP5* rs3736228**	C/C	76.0	0.59	80.3	0.62	1	NS ^a^
	C/T	24.1		18.0		1.4 (0.7–2.8)	
	T/T	0.0		1.6		-	

The most frequent homozygous genotype was taken for reference genotype, the OR values presented are calculated for responders *vs*. non-responders groups. Non-corrected nominal P-values are shown. Bold values are significant after Bonferroni correction.

^a^ At least one genotype frequency is less than 1

^b^ Yates' correction is employed

^с^ Dominant model of inheritance used.

The genotype frequencies of all analyzed SNVs were not significantly different from Hardy-Weinberg equilibrium at 5% level. Comparing the genotype frequencies between responders and non-responders groups, statistically significant differences after Bonferroni correction were observed for *PTH*, *FDPS*, and *GGPS1* gene variants (Table 2).

For the *SOST* rs1234612 variant, we found an association with a favorable response to BPs therapy for the bearers of heterozygous genotype C/T (OR = 0.4; 95% CI 0.2–0.8; P = 0.016), but this association was not significant after Bonferroni correction. Since minor C-allele frequency of *SOST* rs1234612 was low, using dominant model of inheritance, we found that C/C+C/T genotype carriers were under-represented in non-responders compared with the individuals with T/T genotype (OR = 0.4; 95% CI 0.2–0.8, P = 0.004).

The *PTH* rs7125774 C/C genotype carriers were statistically significantly over-represented in responders (OR = 0.2; 95% CI 0.1–0.6; P = 0.0009). Alternatively, the frequency of the T/T genotypes was higher in non-responders group.

The *FDPS* rs2297480 G/G genotypes were statistically significantly over-represented in non-responders group (OR = 29.3; 95% CI 3.6–241.0, P = 2.2×10^−7^), suggesting their negative effect on response to BPs therapy. Since the frequency of *GGPS1* rs10925503 MAF (T allele) was very low, we merged C/T and T/T genotype carriers into one group to be compared with C/C genotype carriers (dominant model of inheritance). The data shows that the frequency of the combined C/T+T/T is statistically significantly higher in non-responders group (OR = 2.9; 95% CI 1.4–5.8, P = 0.003).

However, there was no significant association of *FGF2* rs6854081 or *LRP5* rs3736228 gene variants with the response to BPs treatment.

**Fig 1:**
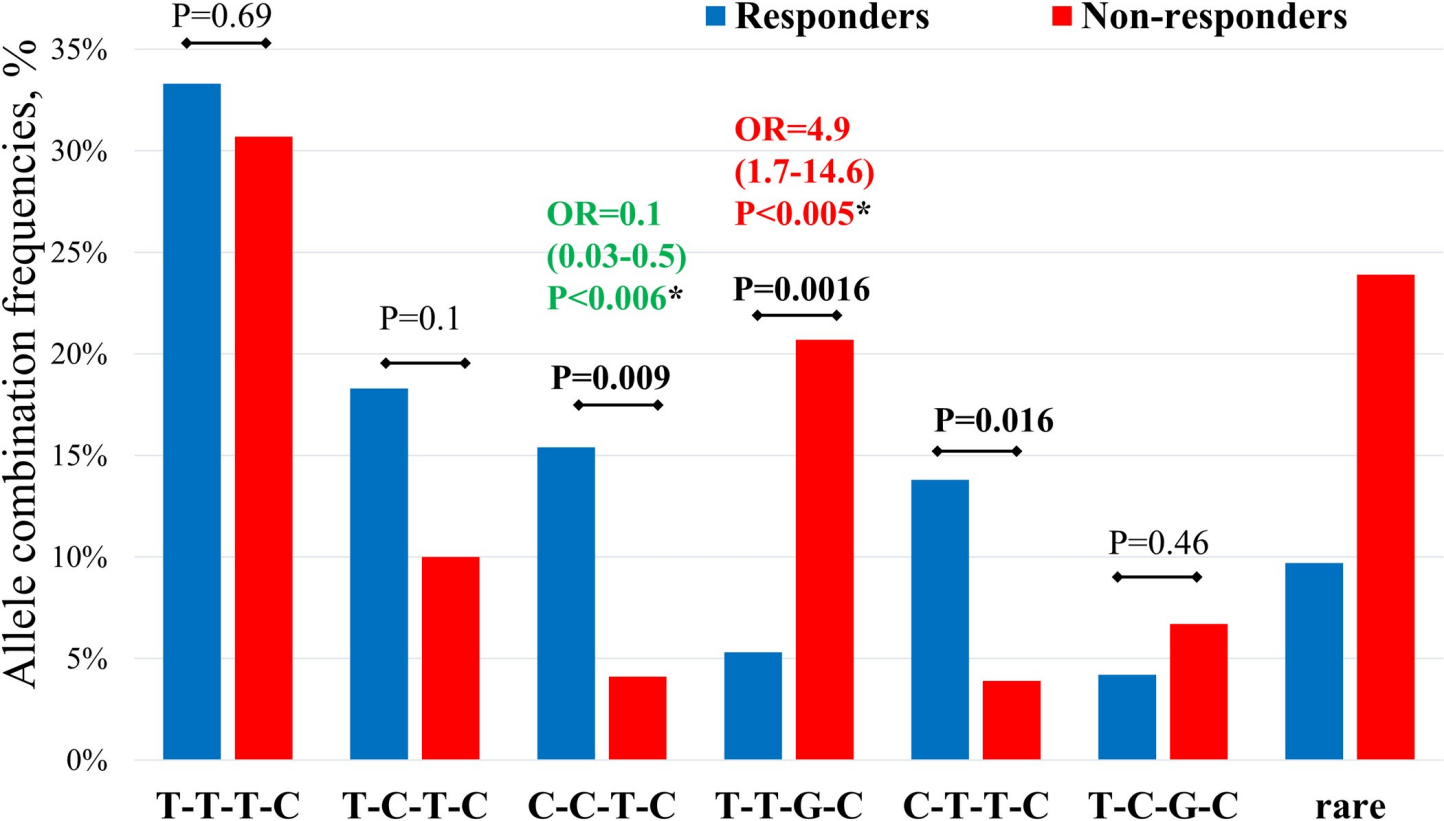
The comparison of frequencies of estimated allelic combinations, constructed from *SOST*, *PTH*, *FDPS*, and *GGPS1* gene variants, in responders and non-responders groups of patients with postmenopausal osteoporosis after 12 months to treatment with BPs. (*)–compared with reference combination.

In a further work, association of allelic combinations of *SOST*, *PTH*, *FDPS*, and *GGPS1* gene variants with response to BPs therapy have been analyzed (Fig 1). The data on *FGF2* and *LRP5* gene variants were removed from further analysis due to the absence of significant association.

Six allelic combinations with the total frequency greater than 5% were constructed from the genotyping results in responders and non-responders. Presence of these 6 most common combinations was found in 85.2% of all subjects. Statistically significant differences between analyzed groups were revealed in the global distribution of allelic combinations (global P-value <0.0001), suggesting an association of the risk of negative response to BPs therapy with the analyzed SNVs frequencies.

The most frequent allelic combination was T-T-T-C (total frequency 32.5%), and it was almost similarly represented in both groups of patients (33.3% responders and 30.7% non-responders, P = 0.66). The frequency of the C-C-T-C combination, generated from three alleles, predisposing to a positive response to BPs treatment (total frequency– 12.2%), was significantly over-represented in the responders group compared with the non-responders group (15.4% and 4.1%, respectively, P = 0.009). For the carriers of this combination, the probability of positive response to BPs therapy was statistically significantly higher when compared with the reference combination (OR = 0.1, 95% CI 0.02–0.5, P = 0.006). In contrast, for the carriers of T-T-G-C combination (11.7% total, 5.3% responders, 20.7% non-responders, P = 0.002), constructed from three alleles of resistance to BPs therapy, the negative response risk was statistically significantly increased (OR = 4.9, 95% CI 1.7–14.6, P = 0.005).

**Fig 2:**
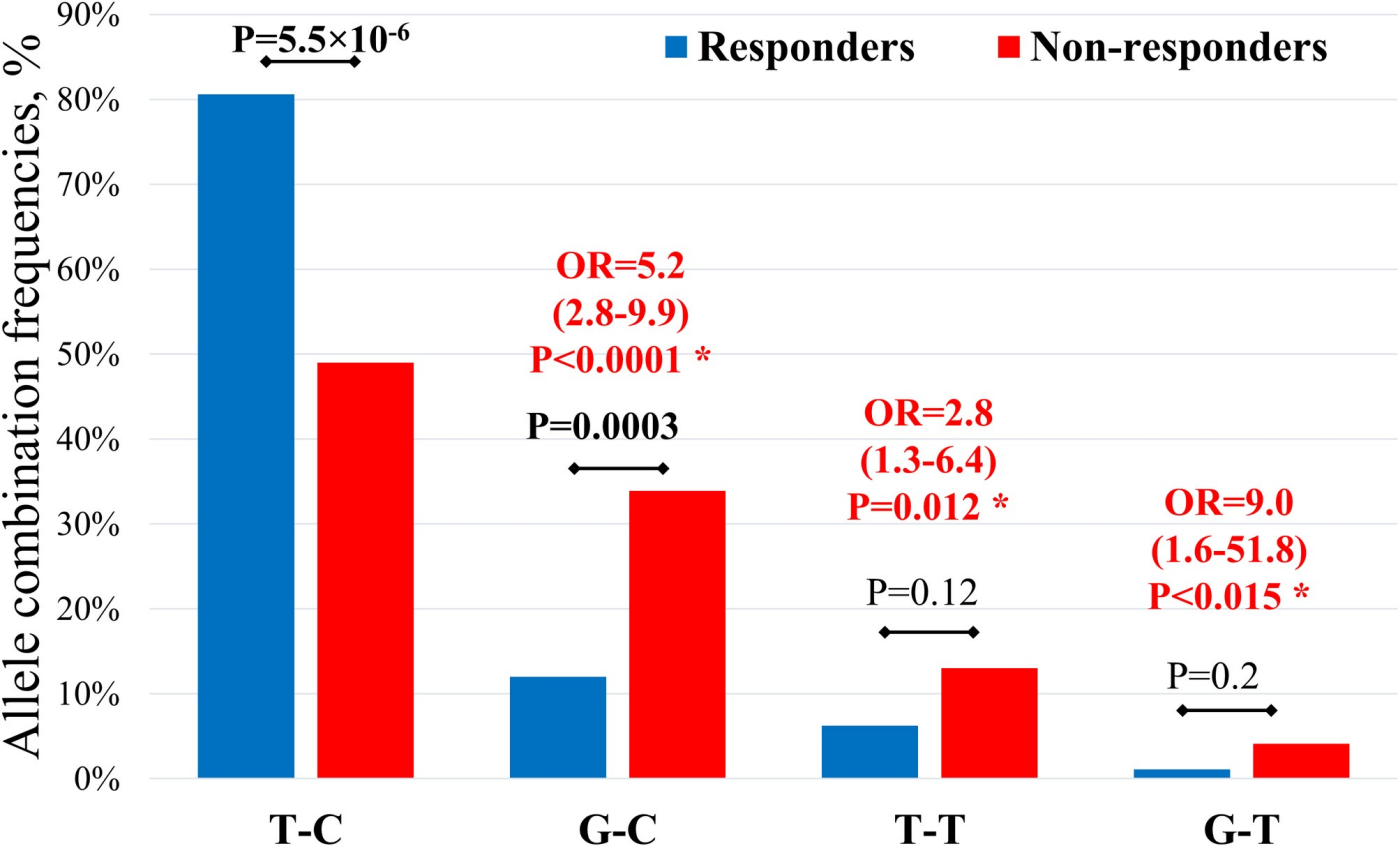
The comparison of frequencies of estimated allelic combinations, constructed from *FDPS* and *GGPS1* gene variants, in responders and non-responders groups of patients with postmenopausal osteoporosis after 12 months to treatment with BPs. (*)–compared with reference combination.

The results of analysis of inferred allelic combinations frequency of *FDPS* and *GGPS1* genes in patients with 12+ months of antiresorptive therapy are presented in Fig 2.

The most frequent is the combination of wild-type T-C alleles (total frequency– 68.9%), which is significantly over-represented in patients with positive response to BPs treatment (80.6% of responders and 49.0% of non-responders, P = 5.5×10^−6^). For the carriers of G-C, T-T and G-T allelic combinations, the risk of negative response for BPs treatment was significantly higher compared with reference combination (OR = 5.2, 2.8, and 9.0, respectively, P<0.015 in all cases). The quantities of careers with these three combinations were under-represented in responders group.

## Discussion

The results of this study show the possible role of allelic combinations of *SOST* rs1234612, *PTH* rs7125774, *FDPS* rs2297480, and *GGPS1* rs10925503 gene variants in the individual response to BPs treatment in women with postmenopausal osteoporosis.

Osteoporosis is an important health problem among postmenopausal women. The effects of antiosteoporotic treatment are determined by bone remodeling processes, involving resorption by osteoclasts and formation by osteoblasts. Long-term treatment is used in patients with osteoporosis, and BPs are the most commonly prescribed medications. However, in some patients this therapy is not effective, cause different side effects. Unfortunately, at least one year of therapy is required to identify and confirm ineffectiveness of BPs therapy on bone mineral density. Among other factors, the response to BPs therapy has a strong hereditary component among other factors. Therefore, elucidating the genetic factors, involved in antiosteoporotic treatment, is very important and will help substantially improve effectiveness of therapy.

Since osteoporosis is strongly associated with genetic factors, there is a growing interest in elucidating the target population with high fracture risk for preventive strategies. On the other hand, there is a clear need for more accurate therapeutic approach, as the acquisition of BMD is a long-term process influenced by many factors and it would be reasonable to target pharmacological treatment in compliance to the personalized characteristics of patients, including genetic ones. However, there are only a few studies conducted in the field of pharmacogenetics of osteoporosis, and they mostly were performed using single candidate gene variants analysis and therefore do not assess the complexity of this multifactorial condition.

In previous studies analysis of genes, involved in bone loss control, mevalonate pathway, farnesyl diphosphate synthase gene, geranylgeranyl diphosphate and some others, was performed [12–20]. Results of these studies assume that genotyping of patients with osteoporosis could be promising tool to identify the category of subjects who are most likely would be responders to antiosteoporotic therapy. Nevertheless, most of these studies have certain limitations, such as insufficient sample size, differences between ethnic groups, sampling errors, influence of confounding factors and others. Despite all the progress in the field of pharmacogenetics in recent years, however, up to date only a few number of SNVs have been identified as potentially associated with response to antiosteoporotic treatment. In present study, we analyzed SNVs and their allelic combinations to evaluate the association of gene variants, involved in bone remodeling and mevalonate pathway, with the response to treatment with BPs.

Even though BPs have been demonstrated to be effective in reducing bone fracture risk in postmenopausal osteoporotic women, about 26–53% of osteoporotic patients have poor response to BPs treatment [11]. In our study, a total of 79 women (39.3%) were included to non-responders group. There was no heterogeneity in the therapeutic effect depending on type of aminobisphosphonate used. The patients with the change of LS BMD value below the LSC level were removed from the study according to ISCD recommendations [23], although in several studies at least patients with BMD level stabilization (increase below LSC level after antiresorptive therapy) were considered as responders [12,14–16,25–27]. Allele and genotype frequencies of analyzed SNVs in our cohort were similar to those of other Caucasian populations and all were in Hardy-Weinberg equilibrium.

The results of our study revealed the association between some genetic markers and response to antiosteoporotic treatment in women with postmenopausal osteoporosis. After analysis of the association of single polymorphic variants of genes involved in the metabolism of BPs, four informative genetic markers related to the effectiveness of antiresorptive therapy were identified: *SOST* rs1234612, *PTH* rs7125774, *FDPS* rs2297480, *GGPS1* rs10925503.

The *SOST* gene encodes a protein sclerostin, an inhibitor of the canonical Wnt-signaling pathway. The rs1234612 variant is located in the 9.7kb region upstream and is revealed as transcription factor binding site, potentially influencing the expression level of sclerostin, as well as bone formation and BMD level. A study of 639 Chinese women with postmenopausal osteoporosis previously showed that *SOST* polymorphic variant correlates with change in the BMD level in the lumbar spine and femoral neck after 12 months of alendronate therapy (P<0.05) [26]. However, considering that the frequencies of *SOST* rs1234612 in Chinese are different from those in European, it was important to analyze the correlation of this SNV and response to BPs in other populations.

To the best of our knowledge, there were no recent publications on the association of the polymorphic variant *PTH* rs7125774 with the effectiveness of antiresorptive therapy, only one genome-wide association study showed statistically significant association of this genetic marker with the level of femoral neck BMD level [28]. Prolonged BPs therapy may lead to excessive activity of parathyroid glands and stimulation of bone resorption. Considering that SNV rs7125774 is located at approximately 100 *kb* upstream of the *PTH* gene, it potentially might regulate *PTH* gene expression through intergenic transcription and, according to our data, may influence the response of BPs treatment, although the actual molecular mechanism requires further investigation in order to understand whether the correlation is caused by the effects of genetic variance on bone turnover or includes BPs metabolism pathways. The revealed highly significant association of *PTH* rs7125774 with effectiveness of BPs therapy should be considered with caution and has to be elucidated in further studies.

Found in our work association of the *FDPS* gene with pronounced response to BPs treatment is not unexpected, since many previous studies on other populations have shown that various polymorphic variants of this gene, as well as the *GGPS1* gene determine the effectiveness of osteoporosis treatment with various BPs [14–16,29]. In general, for the bearers of *FDPS* rs2297480 G-allele or G/G-genotype, the risk of negative response to BPs therapy is increased. At the same time, it is worth noting that in previously published studies the ambiguous results of the association of *FDPS* and *GGPS1* gene variants with antiresorptive therapy of osteoporosis were obtained. The data varied in representatives of different nationalities and in some cases were associated with ethnic characteristics [14,16,29]. This is primarily due to the fact that molecules that are encoded by the *FDPS* and *GGPS1* genes are important enzymes of isoprenoid biosynthesis and serve as primary targets for BPs. Aminobisphosphonates inhibit the farnesyl pyrophosphate synthase step in the mevalonate pathway, thereby modifying the isoprenylation of guanosine triphosphate binding proteins [29]. The results of the analysis of allelic combinations of *FDPS* and *GGPS1* genes, suggesting that a combination of unfavorable alleles dramatically increases the risk of negative response to BPs therapy are of a particular interest. *FDPS* rs2297480 is located in the 5’ region of the gene, 778 bp upstream of the translation site, and may substantially influence gene expression. Thus, it can be assumed that the studied polymorphic gene variants can change the transcription level of *FDPS* or *GGPS1* genes, which in turn can lead to changes in serum concentration of the corresponding molecules and, as a consequence, affect the effectiveness of therapy.

No statistically significant association with the efficacy of BPs therapy has been shown for the gene encoding the second (main) fibroblast growth factor *FGF2* rs6854081. For this genetic marker, as well as for the *PTH* gene, only an association with a femoral neck BMD level was shown, but not with response to BPs treatment [30]. *FGF2* rs6854081 is located at microRNA binding site and therefore may influence the inhibition of protein translation.

The most unexpected result obtained in our study is the absence of statistically significant association of the genetic marker *LRP5* rs3736228 with the response to BPs treatment. Studies on various populations have shown that this missense substitution is associated with both the risk of developing osteoporosis [13,31] and with the effectiveness of its antiresorptive therapy with BPs [32]. It is located in exon 18 of *LRP5* gene and leads to alanine substitution by valine (p.Ala1330Val) in the extracellular region of LPR5, responsible for mediating the interactions of LRP5 and its ligands. According to the literature, T-allele of the polymorphic variant *LRP5* rs3736228 was significantly associated with a weak response to alendronate therapy compared with the carriers of C-allele in Chinese postmenopausal women with OP [32]. In contrast, and in agreement with our results, no association was reported for *LRP5* rs3736228 with osteoporotic therapy in patients from UK [33]. The lack of statistically significant association in our study may be due to the low frequency of T-allele in the examined groups of patients and/or background genetic differences in the populations. As osteoporosis is a complex disease influenced by multiple factors, these inconsistent results could mainly arise from the difference in population selection, ethnicity, sample size, age, and others.

Further comprehensive analysis of allelic combinations was performed to increase the statistical power of the study and to reveal potential gene-gene interactions. According to the results of the analysis of the association of single polymorphic variants of genes involved in the metabolism of BPs, four informative genetic markers statistically significantly associated with the effectiveness of antiresorptive therapy were identified: *SOST* rs1234612, *PTH* rs7125774, *FDPS* rs2297480, *GGPS1* rs10925503.

From the results presented in Fig 1, the same frequency of the reference T-T-T-C combination in both groups of patients is noteworthy (the difference is not significant). This may be explained by the fact that this allelic combination consists of half of the alleles that predispose to resistance and half of the alleles that predispose to sensitivity to BPs therapy. At the same time, the frequency of the allelic combination of T-T-G-C, where resistance alleles predominate, predisposes to an adverse effect on therapy and is more common in the non-responders group. An inverse pattern is found for the C-C-T-C combination. Thus, *SOST* rs1234612, *PTH* rs7125774, *FDPS* rs2297480, *GGPS1* rs10925503 are individually associated with the effectiveness of antiresorptive therapy to some extent, but the most consistent association was revealed in complex allelic combination analysis.

The results of the analysis of the frequencies of allelic combinations suggest a strong pharmacogenic influence and will help to evaluate the possible response to BPs treatment of osteoporosis in advance using genetic testing and, when identifying a large number of resistance alleles, recommend the selection of alternative treatment methods. Taking into consideration that genetic factors may explain up to 95% of the difference in response to antiosteoporotic agents [34], the findings highlight the importance of used genetic approach of bone turnover single gene variants and their allelic combinations identification for pharmacogenetics of BPs therapy of osteoporosis. We suggest repetition of this work in other studies and further implementation as a strategy for personalized antiresorptive therapy of bone metabolism disorders.

## Conclusions

In conclusion, we reported for the first time the importance of identified allelic combinations of *SOST* rs1234612, *PTH* rs7125774, *FDPS* rs2297480, and *GGPS1* rs10925503 gene variants for evaluation of the individual resistance or sensitivity to BPs treatment of osteoporosis. Pharmacogenetic studies of OP are very important for personalized medicine, as they will help to predict the effect of antiresorptive therapy.
